# Risk factors associated with relapse of adult-onset Still disease in Korean patients

**DOI:** 10.1097/MD.0000000000023579

**Published:** 2020-12-11

**Authors:** Ji-Hyoun Kang

**Affiliations:** Division of Rheumatology, Department of Internal Medicine, Chonnam National University Medical School & Hospital, Gwangju, Republic of Korea.

**Keywords:** adult-onset Still disease, arthritis, lymphadenopathy, relapse

## Abstract

Approximately 30% to 40% of all patients with adult-onset Still disease (AOSD) experience relapses, sometimes presenting as chronic damage, and these events can subsequently increase the morbidity and mortality in patients with AOSD. However, few studies are investigating the factors related to relapse in such patients. Therefore, this study aimed to explore the risk factors associated with relapse of AOSD.

This cohort study enrolled 112 AOSD patients who satisfied the Yamaguchi criteria and obtained available data from Chonnam National University Hospital. The demographic, clinical, and laboratory data as well as treatment history of the patients from January 2008 to December 2019 were retrospectively reviewed. Relapse events were defined as the presence of one or more recurrent events. Multivariate logistic regression analysis was performed to investigate the possible risk factors for relapse.

During a mean follow-up of 103.3 months, 47 of 112 patients (41.9%) developed a relapse. According to the results of multivariate logistic regression analysis, arthritis (odds ratio [OR] = 19.530, 95% confidence interval [CI]: 5.047–75.582, *P* < .001) and lymphadenopathy (OR = 6.539, 95% CI: 2.329–18.358, *P* < .001) predicted the development of recurrent events in patients with AOSD.

Patients with AOSD had frequent relapses during the clinical course of their disease. Risk factors associated with flares were the presence of arthritis and lymphadenopathy.

## 1 Introduction

Adult-onset Still disease (AOSD) is an uncommon systemic autoinflammatory disease of unclear etiology characterized by high spiking fever, arthritis, and skin rash.^[1–4]^ There are 3 different disease course patterns in AOSD: monocyclic pattern with 1 episode of relapse; polycyclic pattern with multiple relapses and wax and wane courses; and chronic pattern with unremitting activation of disease.^[5–7]^ Among those patterns, approximately 30% to 40% of all patients with AOSD develop a recurrent or polycyclic pattern characterized by relapses, sometimes presenting as chronic damages. Some patients with AOSD showed a poor response to steroid treatment. In addition, relapses can subsequently increase morbidity and mortality in patients with AOSD. Especially, Asian patients with AOSD were reported to have a higher in-hospital mortality rate.^[8]^ According to a study by Zeng et al who reviewed 61 cases of AOSD, patients with >2-year disease duration have shown mortality of 12%.^[9]^ In a long-term follow-up study of 8 patients with AOSD, 2 died.^[10]^ However, despite the necessity of AOSD research, only a few studies exist on the factors related to relapse in AOSD patients, and the previous studies investigated only a small population.

Therefore, this study aimed to identify the risk factors related to the occurrence of relapse in AOSD patients.

## 2 Patients and methods

### 2.1 Study populations

This study enrolled 112 patients with AOSD who satisfied the Yamaguchi criteria^[11]^ and their clinical data were obtained from Chonnam National University Hospital (CNUH). All patients charts were reviewed retrospectively. Relapse events were defined as the presence of one or more recurrent events. Patients diagnosed with a disease other than AOSD during follow-up or who were lost to follow-up were excluded. The patients were divided into 2 groups depending on whether they had experienced relapse or not. Sociodemographic, clinical, and laboratory data, and history of treatment strategies were obtained. Demographic characteristics included age at onset, sex, and disease duration. Clinical manifestations such as high spiking fever, skin rash, arthritis, lymphadenopathy, liver dysfunction, sore throat, myalgia, splenomegaly/hepatomegaly, pericarditis, pleuritis, acute respiratory distress syndrome (ARDS), hemophagocytic lympho-histiocytosis (HLH), and macrophage activation syndrome (MAS) were assessed. Laboratory findings, including a complete blood count, liver function tests, ferritin level, erythrocyte sedimentation rate (ESR), C-reactive protein (CRP), and neutrophil counts were assessed, as well as the levels of rheumatoid factor, anti-cyclic citrullinated peptide (CCP), and anti-nuclear antibody (ANA). This study also evaluated treatment strategies, including prednisolone dosage (daily mean dose, cumulative dose, and maximal dose) and use of nonsteroidal anti-inflammatory drugs (NSAIDs), disease-modifying anti-rheumatic drugs (DMARDs) (methotrexate, azathioprine, and calcineurin inhibitor), intravenous immunoglobulin (IVIG), tumor necrosis factor (TNF) inhibitor, anakinra, and tocilizumab. This study was approved by the Institutional Review Board of CNUH (CNUH-2020-043). Because of the retrospective study design, the requirement for informed consent from patients was waived.

### 2.2 Statistical analysis

All statistical analyses were performed using SPSS for Windows (version 21.0; SPSS, Chicago, IL, USA). Values are expressed as means ± standard deviations for continuous variables and percentages for categorical variables. To compare the differences between 2 groups, continuous variables were estimated using the Mann–Whitney test and the Kruskal-Wallis test. Categorical variables were analyzed using a Chi-Squared test. To identify the factors associated with the occurrence of relapse in patients with AOSD, univariate and multivariate logistic regression analyses were performed to calculate odds ratios (OR) for dependent variables, which were calculated with a 95% confidence interval (95% CI). A *P* < .05 was considered statistically significant.

## 3 Results

Among the 112 patients, 47 patients (41.9%) relapsed during a mean follow-up duration of 103.3 months. Patients mean age was 45.0 ± 13.7 years, and 65.2% (73 patients) of the total patients were women.

Table 1 shows the baseline characteristics between the relapse and non-relapse groups. The group with relapse had a higher proportion of patients with arthritis (*P* < .001), lymphadenopathy (*P* < .001), pericarditis (*P* = .035), and pleuritis (*P* = .007) at baseline than the group without relapse. There was no significant difference in the onset age, sex, disease duration, and clinical manifestations such as fever, rash, sore throat, and liver dysfunction between the relapse and non-relapse groups.

**Table 1:** Baseline characteristics between the relapse and non-relapse groups.

	Relapse (N = 47)	Non-relapse (N = 65)	*P* value
Age at onset (years)	46.3 ± 15.2	43.9 ± 14.8	.533
Female sex	32 (68.1)	41 (63.1)	.583
Disease duration (months)	105.0 ± 75.0	96.1 ± 75.3	.471
Spiking fever	46 (97.9)	65 (100.0)	.237
Skin rash	41 (87.2)	57 (87.7)	.942
Arthritis	44 (93.6)	28 (43.1)	<.001
Lymphadenopathy	29 (61.7)	16 (24.6)	<.001
Liver dysfunction	27 (57.4)	28 (43.1)	.133
Sore throat	17 (36.2)	29 (44.6)	.370
Myalgia	39 (83.0)	44 (67.7)	.068
Splenomegaly	5 (10.6)	3 (4.6)	.222
Hepatomegaly	1 (2.1)	2 (3.1)	.759
Pericarditis	5 (10.6)	1 (1.4)	.035
Pleuritis	7 (18.4)	1 (1.4)	.007
ARDS	2 (4.3)	0 (0)	.174
Malignancy	1 (2.6)	0 (0)	.339
HLH	2 (4.3)	5 (7.7)	.458
MAS	1 (2.6)	0 (0)	.339

ARDS = acute respiratory distress syndrome, HLH = hemophagocytic lympho-histiocytosis, MAS = macrophage activation syndrome.

The comparison of the laboratory findings between the 2 groups is shown in Table 2. The relapse group showed a higher ferritin level (*P* < .001) and white blood cell (WBC) counts (*P* = .003) than the non-relapse group. The rheumatoid factor, anti-CCP, ANA, ESR, and CRP levels were not significantly different between the 2 groups.

**Table 2:** Laboratory findings between the relapse and non-relapse groups.

	Relapse (N = 47)	Non-relapse (N = 65)	*P* value
Rheumatoid factor	4 (8.5)	3 (4.6)	.401
Anti-CCP antibodies	2 (5.3)	0 (0)	.093
ANA	6 (15.8)	16 (21.6)	.119
Ferritin	16741.8 ± 18177.2	9014.8 ± 13755.4	<.001
White blood cell counts	18847.2 ± 11013.1	12715.4 ± 6098.7	.003
ESR	67.2 ± 31.3	65.3 ± 35.9	.220
CRP	13.5 ± 9.4	10.4 ± 7.5	.055
Neutrophil counts	87.0 ± 5.8	83.7 ± 8.5	.334

ANA = anti-nuclear antibody, Anti-CCP = anti-cyclic citrullinated peptide antibody, CRP = C-reactive protein, ESR = erythrocyte sedimentation rate.

Table 3 shows the treatment strategies between the 2 groups. The patients with relapse were more frequently prescribed a higher dosage of mean prednisolone (maximum dose used during the treatment period) (*P* = .023) and maximal prednisolone (*P* = .028) than those without a relapse. The relapse group had a higher proportion of patients prescribed CNI (*P* = .010) and tocilizumab (*P* = .017) than the non-relapse group. There was no difference in the cumulative prednisolone dosage, and the proportion of patients prescribed NSAIDs, methotrexate, azathioprine, IVIG, and TNF inhibitor between the 2 groups.

**Table 3:** Treatment strategies between the relapse and non-relapse groups.

	Relapse (N = 47)	Non-relapse (N = 65)	*P* value
NSAIDs (%)	46 (97.9)	62 (95.4)	.484
Mean prednisolone (mg/kg/day)	0.13 ± 0.06	0.18 ± 0.14	.023
Maximal prednisolone (mg/kg/day)	1.42 ± 0.89	1.04 ± 0.76	.028
Cumulative prednisolone (mg/kg/day)	0.11 ± 0.10	0.12 ± 0.14	.549
DMARDs			
Methotrexate (%)	36 (76.6)	46 (70.8)	.492
Azathioprine (%)	4 (8.5)	6 (9.2)	.895
CNI (%)	25 (53.2)	19 (29.2)	.010
IVIG	1 (2.1)	0 (0)	.237
TNF inhibitor (%)	1 (2.6)	0 (0)	.339
Anakinra (%)	1 (2.6)	0 (0)	.237
Tocilizumab (%)	10 (21.3)	4 (6.2)	.017

CNI = calcineurin inhibitor, DMARDs = disease modifying anti-rheumatic drugs, IVIG = intravenously immunoglobulin, NSAIDs = non-steroidal anti-inflammatory drugs, TNF = tumor necrosis factor.

As a result of the univariate logistic regression analysis, arthritis (OR = 19.381, 95% CI: 5.452–68.902, *P* < .001), lymphadenopathy (OR = 4.934, 95% CI: 2.184–11.148, *P* < .001), pleuritis (OR = 11.200, 95% CI: 1.328–94.455, *P* = .026), WBC count >10,000/μl (OR = 2.472, 95% CI: 1.021–5.983, *P* = .045), CNI use (OR = 2.751, 95% CI: 1.257–1.349, *P* = .011), cumulative prednisolone dosage (OR = 1.184, 95% CI: 1.040–1.349, *P* = .011), and tocilizumab use (OR = 4.122, 95% CI: 1.205–14.092, *P* = .024) were significant predictors of relapse in AOSD patients (Table 4). In the multivariable logistic regression analysis, arthritis (OR = 19.530, 95% CI: 5.047–75.582, *P* < .001) and lymphadenopathy (OR = 6.539, 95% CI: 2.329–18.358, *P* < .001) were significant predictors of the development of recurrent events in patients with AOSD (Table 4).

**Table 4:** Univariate and multivariate logistic regression analyses of the factors associated with relapse.

	Univariate regression		Multiple regression	
Variable	Odds ratio (95% CI)	*P* value	Odds ratio (95% CI)	*P* value
Arthritis	19.381 (5.452–68.902)	<.001	19.530 (5.047–75.582)	<.001
Lymphadenopathy	4.934 (2.184–11.148)	<.001	6.539 (2.329–18.358)	<.001
Pleuritis	11.200 (1.328–94.455)	.026	3.618 (0.197–66.348)	.386
Pericarditis	7.619 (0.860–67.537)	.068		
Ferritin>2000 ng/ml	1.675 (0.755–3.714)	.204		
WBC count>10,000/μl	2.472 (1.021–5.983)	.045	1.419 (0.422–4.773)	.572
CNI	2.751 (1.257–6.023)	.011	1.960 (0.685–5.614)	.210
Tocilizumab	4.122 (1.205–14.092)	.024	4.332 (0.921–20.371)	.063

CI = confidence interval, CNI = calcineurin inhibitor, WBC = white blood cell.

## 4 Discussion

This study demonstrated that arthritis and lymphadenopathy at baseline were significantly associated with recurrent events during the clinical course of their disease in patients with AOSD.

Patients with AOSD who had arthritis at baseline showed significant relapses. Regardless of high spiking fever, patients with arthritis also showed a higher ferritin level than those without arthritis. In addition, patients with arthritis were more frequently prescribed mean and maximal prednisolone dosage than those without arthritis. Similarly, Kalyoncu et al reported that male sex, delayed diagnosis of >6 months, failure of initial treatment, and arthritis were significant factors related to the chronic course of AOSD.^[12]^ In addition, Ichida et al found that AOSD patients with rheumatoid arthritis (RA) subtype showed involvement of multiple joints, and all of these patients showed a deterioration of multiple joints.^[13]^ In this study, AOSD patients with arthritis showed multiple joint involvements, and several patients have shown erosive changes. The musculoskeletal manifestations usually follow the development of the systemic manifestations, and chronic erosive changes have been found in approximately one-third of AOSD patients.^[14]^ Some of the patients with AOSD experienced severe joint damages. Multiple joint damages can lead to functional impairment and low quality of life. Therefore, because prednisolone treatment may be insufficient in patients with arthritis, physicians may consider intensive treatments, such as administration of DMARDs or biologics to these patients in the earlier phase. Early induction of DMARDs or biologics can also have a role as sparing agents of corticosteroids dosage.

The present study demonstrated that AOSD patients with lymphadenopathy frequently experienced recurrent events. A previous study by Sun et al reported that the interleukin-10 level was increased in AOSD patients who had lymphadenopathy and were significantly associated with the disease activity ^[15]^ in patients during the earlier phase. Moreover, Chi et al found that interleukin-37 levels were correlated to the disease activity in AOSD patients who had lymphadenopathy.^[16]^ Lymphadenopathy is one of the major symptoms in AOSD and is closely related to disease activity. Like prior studies, lymphadenopathy may contribute to relapses due to the high level of inflammatory cytokines observed in the patients. According to a previous study that analyzed the predictive factors of developing MAS in AOSD patients, lymphadenopathy was significantly associated with MAS occurrence.^[17]^ Therefore, when diagnosing AOSD, the patients should be assessed for the presence of lymphadenopathy at baseline in clinical practice to prevent any relapse or complication.

Moreover, the maximal and mean doses of prednisolone were associated with the relapse of AOSD patients in this study. Also, patients with higher disease severity were prescribed a higher percentage of tocilizumab and calcineurin inhibitors. The dosage of prednisolone and immunosuppressors in the clinical course of AOSD is proportional to the disease activity and its clinical symptoms. Physicians tend to consider more intensive treatments, particularly in patients with severe symptoms. The more the symptoms, the higher the dosage of medication. Thus, this finding may be natural in AOSD.

There are some limitations to this study. First, this study had a retrospective design; thus, I could not identify the causal relationship between the risk factors and relapses. Second, the enrolled patients were followed up at a single center; thus, they might not represent all AOSD patients with various characteristics.

In conclusion, the risk factors associated with flares were the presence of arthritis and lymphadenopathy at baseline in patients with AOSD. The results of this study suggest the necessity for intensive management of relapse in these patients. Further prospective studies with a large sample size will be needed to investigate the risk factors for relapses in AOSD patients clearly.

## Acknowledgments

I would like to thank the patients who offered their information in this study.

## Author contributions

**Conceptualization:** Ji-Hyoun Kang.

**Data curation:** Ji-Hyoun Kang.

**Formal analysis:** Ji-Hyoun Kang.

**Investigation:** Ji-Hyoun Kang.

**Methodology:** Ji-Hyoun Kang.

**Project administration:** Ji-Hyoun Kang.

**Resources:** Ji-Hyoun Kang.

**Software:** Ji-Hyoun Kang.

**Supervision:** Ji-Hyoun Kang.

**Validation:** Ji-Hyoun Kang.

**Visualization:** Ji-Hyoun Kang.

**Writing – original draft:** Ji-Hyoun Kang.

**Writing – review & editing:** Ji-Hyoun Kang.
